# A Case Report of a Novel Harm Reduction Intervention Used to Detect Opioid Overdose in the Emergency Department

**DOI:** 10.5811/cpcem.2020.7.47936

**Published:** 2020-09-18

**Authors:** Kraftin E. Schreyer, Saloni Malik, Andrea Blome, Joseph L. D’Orazio

**Affiliations:** Temple University Hospital, Department of Emergency Medicine, Philadelphia, Pennsylvania

**Keywords:** Harm reduction, opioid use disorder, overdose, opioid overdose

## Abstract

**Introduction:**

As over 130 people die daily from opioid overdose in the United States, harm reduction strategies have become increasingly important. Because public restrooms are a common site for opioid overdose, emergency department waiting room restrooms (EDWRR) should be considered especially high-risk areas.

**Case Report:**

We present the case of a patient found after a presumed opioid overdose in our EDWRR. Staff were alerted to his condition by a reverse motion detector (RMD), and rapidly treated him with naloxone.

**Conclusion:**

The RMD is a novel intervention that can save lives and should be considered in EDs with a high incidence of opioid overdose.

## INTRODUCTION

As over 130 people die daily from opioid overdose in the United States, healthcare facilities have an important role in treating patients with opioid use disorder (OUD).^1^ These patients commonly present to emergency departments (ED) not only for acute overdoses, but also for a variety of health problems that are associated with substance use. Harm reduction techniques are becoming increasingly important in combating OUD. These “interventions aimed at reducing the negative effects of health behaviors without necessarily extinguishing the problematic health behaviors completely” can make a significant public health impact.^2^ In the context of OUD, harm reduction techniques have targeted the negative consequences of opioid use, such as overdose and transmission of hepatitis C and human immunodeficiency virus infection, rather than opioid use itself.^2^–^4^ Such harm reduction strategies include syringe exchange, supervised consumption, bystander naloxone distribution, drug screening services, and opioid maintenance programs.^2^,^3^

People who inject drugs, especially unsheltered individuals, frequently use public restrooms for the relatively clean and private environment while also providing both good lighting and access to tap water.^5^ Overdoses, both fatal and non-fatal, commonly occur in public restrooms in places like restaurants, small businesses, and hospitals.^5^–^7^ While some patients with OUD enter EDs via ambulance or private vehicle after an overdose, some patients enter the ED waiting room for unrelated reasons, and subsequently may use drugs in an ED restroom while waiting to be seen by a provider. Reverse or anti-motion detectors (RMD) have been used successfully to reduce overdose deaths and the harm associated with OUD in the outpatient setting including clinics and supervised injection sites, but to our knowledge there are no reports of implementation in ED waiting room restrooms (EDWRR).^5^,^8^,^9^

There has been an increase in drug-related ED visits from 3.19 per 1000 in 2017 to 7.69 per 1000 visits in 2018 in the City of Philadelphia.^10^ Our ED is located in the Kensington section of Philadelphia, which has been described as the “largest open-air narcotics market for heroin on the East Coast,” and saw the greatest number of nonfatal overdoses and the highest number of naloxone administrations in Philadelphia in 2018.^10^,^11^ An RMD medical alert system Alert1 (AlertOneServices, LLC, Williamsport, PA) was installed in one of our EDWRRs in May 2019.

We present the case of a patient found in our EDWRR after a suspected opiate overdose. Staff were alerted to the patient’s condition by an RMD.

## CASE REPORT

In August 2019, at approximately 6:30 pm, the RMD located in the EDWRR alarmed, prompting nursing, physician, and security staff to respond. A 27-year-old White male with past medical history of OUD and bipolar disorder was found lying on the floor of the restroom, unconscious, apneic, and cyanotic with pinpoint pupils. A syringe was noted to be lying on the floor next to the patient, and he later reported injecting while in the EDWRR.

An ED nurse then obtained intranasal naloxone from the ED Pyxis MedStation (Pyxis Corporation, San Diego, CA) and administered two milligrams intranasally. Within a minute, the patient had an improvement in mental status and respirations. He was observed for several minutes without clinical deterioration. Unfortunately, he refused further care and left the ED without further treatment or monitoring. He subsequently established care with our on-campus, office-based opioid treatment program and started on buprenorphine/ naloxone. At the time of the overdose event, the patient was living on the street and using more than 10 bags of heroin per day. He has since relocated to live with his family and has been stable on buprenorphine/naloxone ever since. He last filled his prescription within a week of the writing of this case report.

## DISCUSSION

This case report demonstrates that RMDs are effective at alerting staff to an unconscious patient in an EDWRR. The staff was able to respond quickly, reverse an opioid overdose with naloxone, and prevent significant morbidity and mortality. While some would argue that reversal of an opioid overdose with naloxone neither prevents a future overdose nor provides long-term treatment for OUD, this patient was able to survive the overdose event to later engage with recovery services. Harm reduction strategies like this are increasingly important during the opioid epidemic as more patients die annually from opioid overdose.

Our ED is a community affiliate of an academic institution that sees an annual volume of about 49,000 patients; 3.5% of visits have a primary diagnosis of non-fatal opioid overdose. On average, 4.8 patients per day present to this ED primarily for non-fatal opioid overdose. It is staffed by two attending physicians, seven nurses, and three security guards, and is equipped with six restrooms; two are located in the main ED waiting room area, two are located in the main ED, and two are located in the separate minor care section of the ED. Both EDWRRs are private, all-gender restrooms.

At our institution, costs for RMD equipment and installation totaled $4,700 and installation took one week. The RMD is set on a timer to trigger when the door to the restroom is closed. After a total time of 120 seconds of no motion, an alarm activates. The alarm consists of both a loud sound and flashing lights. The sound can be heard throughout the ED, and the lights flash in the main ED, the triage area, and in the main waiting room. The alarm can only be turned off by using a key on a wall-mounted console located adjacent to the EDWRR where the RMD is installed. The alarm triggers an immediate response by at least one security guard, one nurse, and one physician to the EDWRR to determine whether further intervention is needed. Upon arrival at the EDWRR, staff knock and verbalize that the door will be opened, to make every effort to protect the privacy of the occupant.

CPC-EM Capsule

What do we already know about this clinical entity?

*As over 130 people die daily from opioid overdose in the United States. Harm reduction strategies have become increasingly important*.

What makes this presentation of disease reportable?

*To our knowledge this is the first report of a reverse motion detector (RMD) used in an emergency department (ED) setting*.

What is the major learning point?

*The RMD is a novel intervention that can save lives and should be considered in EDs with a high incidence of opioid overdose*.

How might this improve emergency medicine practice?

*Installation of RMDs in EDs with a high incidence of opioid overdose is documented here to have saved a life, and may be cost-effective. Although false alarms are common, this novel intervention can save lives*.

In the first four months after installation, the RMD alarmed a total of 367 times. While each alarm did require the response of staff, the response time was brief. This presumably had no impact on the care of other patients in the ED during that time, because there were additional staff members in each of the response groups available to care for other patients. The most common cause of a false alarm was the EDWRR being closed without being occupied. The addition of signage in the waiting room and a mechanism to prop the door open reduced the frequency of false alarms.

While there is literature supporting the use of harm reduction techniques, including RMDs, in public restrooms, to our knowledge this case report is the first evidence supporting the use of an RMD in an ED. In comparison to the cost of the RMD, the potential cost of morbidity and mortality associated with opioid overdose is much greater. Furthermore, this case illustrates the potential for a life-saving incident to begin the process of long-term recovery from OUD.

## CONCLUSION

This case report suggests that reverse motion devices in ED waiting room restrooms can effectively alert staff to an unconscious patient with an opioid overdose. The RMD is a novel, cost-effective intervention that can save lives and should be considered in EDs located in areas with a high incidence of opioid overdose.
